# Design of Low-Noise CMOS Image Sensor Using a Hybrid-Correlated Multiple Sampling Technique

**DOI:** 10.3390/s23239551

**Published:** 2023-12-01

**Authors:** Seung Ju Youn, Su Yeon Yun, Hoyeon Lee, Kwang Jin Park, Jiwon Kim, Soo Youn Kim

**Affiliations:** Department of Semiconductor Science, Dongguk University, Seoul 04620, Republic of Korea

**Keywords:** CMOS image sensor, low dark random noise, adaptive-dual gain ADC, correlated multiple sampling

## Abstract

We present a 320 × 240 CMOS image sensor (CIS) using the proposed hybrid-correlated multiple sampling (HMS) technique with an adaptive dual-gain analog-to-digital converter (ADC). The proposed HMS improves the noise characteristics under low illumination by adjusting the ADC gain according to the incident light on the pixels. Depending on whether it is less than or greater than 1/4 of the full output voltage range from pixels, either correlated multiple sampling or conventional-correlated double sampling (CDS) is used with different slopes of the ramping signals. The proposed CIS achieves 11-bit resolution of the ADC using an up-down counter that controls the LSB depending on the ramping signals used. The sensor was fabricated using a 0.11 μm CIS process, and the total chip area was 2.55 mm × 4.3 mm. Compared to the conventional CDS, the measurement results showed that the maximum dark random noise was reduced by 26.7% with the proposed HMS, and the maximum figure of merit was improved by 49.1%. The total power consumption was 5.1 mW at 19 frames per second with analog, pixel, and digital supply voltages of 3.3 V, 3.3 V, and 1.5 V, respectively.

## 1. Introduction

**Figure 1:**
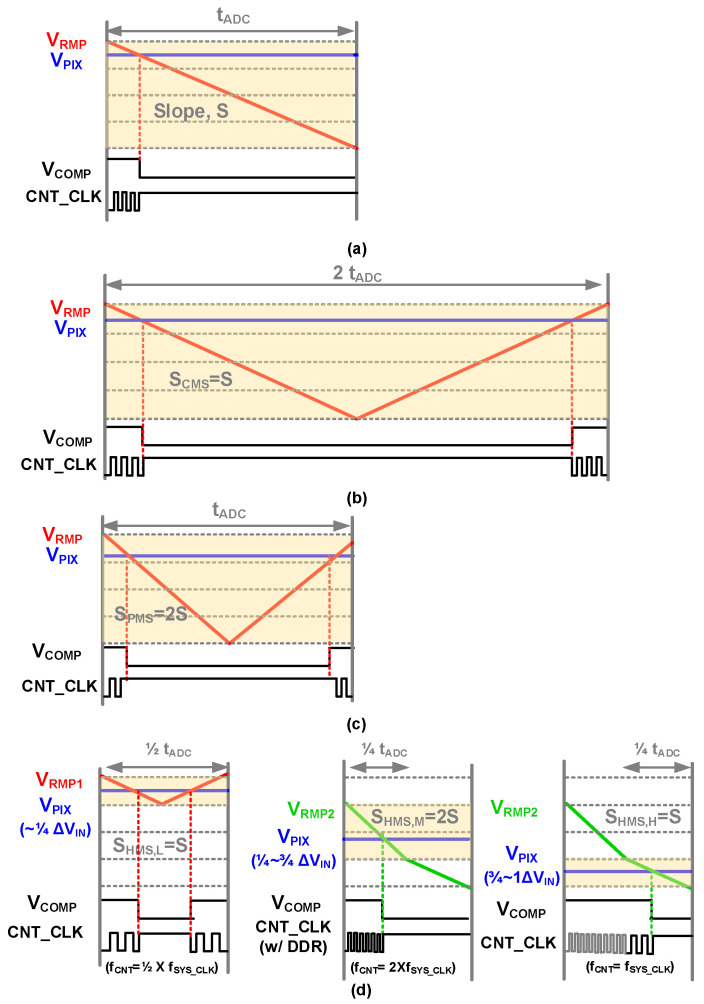
ADC conversion periods of (**a**) conventional CDS, (**b**) CMS, (**c**) pseudo-CMS, and (**d**) the proposed HMS.

As various technologies, such as the Internet of Things (IoT) and self-driving cars, have developed, CMOS image sensors (CISs) go beyond the role of sensing on behalf of the human eye and require the ability to recognize objects, even in situations where it is difficult to observe them with the human eye [1,2,3]. To this end, various circuit techniques have been proposed to obtain high-quality images by increasing the dynamic range or reducing dark random noise (DRN) [4,5,6]. In general, CIS uses a correlated double sampling (CDS) technique to reduce readout noise by sampling the output voltages of the pixel twice. A correlated multiple sampling (CMS) technique that repeats the CDS process several times has been proposed to reduce DRN [7,8,9,10]. Figure 1a,b show the analog-to-digital converter (ADC) conversion periods during signal sampling when using CDS and CMS, respectively. The CMS repeats the ADC *m* times to obtain *m* times more digital output code than the conventional CDS technique. DRN can be reduced by averaging the output code. However, as shown in Figure 1b, the CMS technique repeats the ADC time several times (twice in Figure 1b), which increases the 1-H(horizontal) time and ultimately reduces the frame rate per second (FPS). To solve this problem, a pseudo-CMS (PMS) that maintains the ADC time and adjusts the analog gains by adjusting the slopes of the ramping signals was proposed, as shown in Figure 1c [11]. When the ramping slope for conventional CDS operation is *S*, the slope of PMS, *S_PMS_*, is 2*S* (analog gain = 1/2Χ), and the output code is half that of Figure 1a. To obtain the same output code, the counter clock frequency CNT_CLK should be doubled. However, PMS uses the same CNT_CLK as CDS and adds the number of counting clocks during the up and down operations to obtain the same output code as the CDS technique. In the case of PMS, to generate odd output codes, the maximum voltage of the slope in the upward direction should be V_RMP_-V_LSB_, resulting in a difference in the up- and down-ramping slopes. In addition, because the PMS technique lowers the analog gain, which lowers the ADC resolution by one bit, the quantization noise of the ADC increases. Therefore, to maintain ADC resolution, a high-speed CNT_CLK as high as a multiple of the analog gain is required for a dual-gain-based ramp ADC [12]. 

Figure 1d shows the proposed hybrid-correlated multiple sampling (HMS) technique, which divides the CMS and CDS sections according to illumination. While reducing the DRN with CMS under low illumination, the FPS can be increased under high illumination, which requires a long ADC time by adjusting the analog gain with conventional CDS, called an adaptive dual-gain ADC. When the input is divided into four parts, under low illumination (1/4 of the input), the CMS ramping signal (V_RMP1_) goes down and up only at the 1/4 point. The slope, S_HML_,_L_, is *S*, and the output code is the same as that in Figure 1a. If the input was greater than 1/4, V_RMP2_ was used. When using V_RMP2_, the ramping start voltage of V_RMP2_ is lower than that of V_RMP1_ by 1/4 of ΔV_IN_ (=V_RES_ − V_SIG_), and S_HMS,M_ is *2S*. Unlike [12], a double data rate (DDR) structure is used instead of using 2Χ higher f_CNT_CLK_, leading to the use of CNT_CLK for all operations. Finally, 1/4 of the input part (under high illumination) used the same CNT_CLK and S to achieve a linear ADC transfer curve. Therefore, the HMS proposed for a low-noise CIS operates faster than the techniques mentioned without high-frequency clocks.

**Figure 2:**
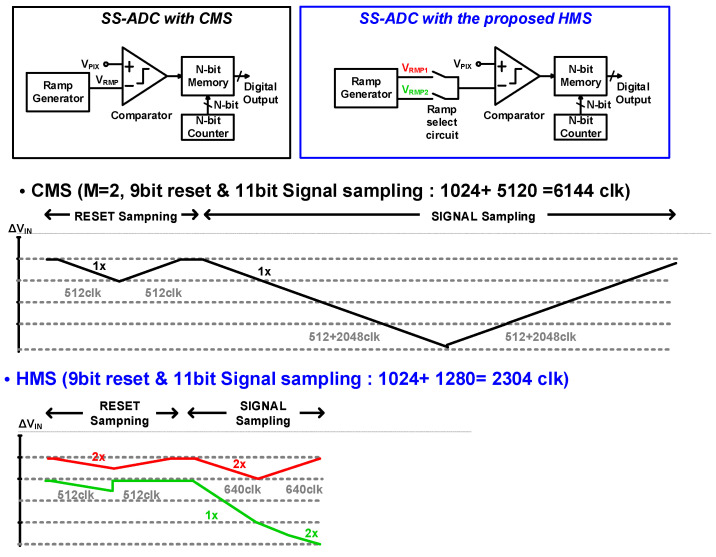
SS-ADC block diagrams and timing diagrams with CMS and the proposed HMS.

Figure 2 compares the detailed SS-ADC timing diagrams of the conventional CMS and the proposed HMS. The CMS shown in Figure 2 requires two sampling times and an ADC time of 6144 clks, which is 2.4 times longer than that of the regular CDS (t_ADC_ = 2560 clk). CMS is performed to reduce the DRN by twice the ADC time. The proposed single-slope ADC (SS-ADC) with an HMS is identical to the existing SS-ADC structure, except for the ramp generator. For HMS operation, two different ramping slopes (V_RMP1_ and V_RMP2_) in the low- and high-illuminance ranges are required. Specifically, 0–1/4 of ΔV_IN_ (low-illuminance range), V_RMP1_ with a doubled analog gain is used; 1/4–3/4 of ΔV_IN_ (middle-illuminance range), V_RMP2_ with an analog gain is used; and in 3/4–1 of ΔV_IN_ (high illuminance range), V_RMP2_ with a doubled analog gain is used to achieve low noise and high frame rates. 

Therefore, the proposed CIS with HMS reduces low-illumination noise through CMS and increases the FPS by adjusting the analog/digital gains. The CMS in this study was implemented through 1-bit shifting after removing the LSB without the additional circuit required by the existing CMS. In addition, the D-CDS time can be reduced without a higher counter-clock frequency with a double data rate (DDR). The remainder of this paper is organized as follows. In Section 2, we describe the detailed operation and implementation of the CIS structure using the proposed HMS. Section 3 presents the measurement results. Finally, the conclusions are presented in Section 4.

## 2. Proposed CIS Structure

**Figure 3:**
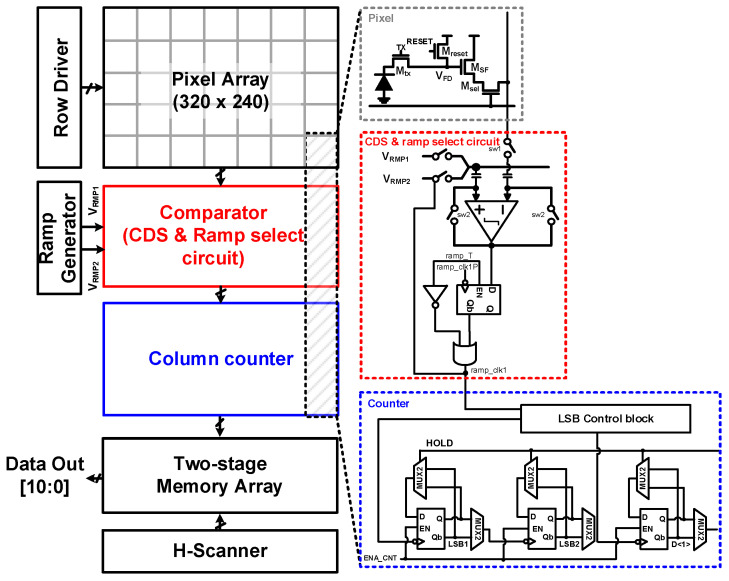
A full block diagram of the proposed CIS.

Figure 3 shows the proposed CIS consisting of an image resolution of 320 × 240 pixels and an 11-bit column-parallel SS-ADC structure using CMS under low illumination and CDS with adaptive-dual gain in middle/high-illumination conditions. The prototype sensor consisted of a pixel array, an 11-bit column counter for the HMS, a ramp-select circuit using feedback to separate low and high incident light levels, and other digital peripheral blocks. In addition, the readout circuit of each column consisted of a comparator, 11-bit two-stage static random-access memory (SRAM), and a column decoder for H-scanning. A two-stage SRAM allows for simultaneous input from the first stage while outputting values from the second stage. Using second-stage SRAM, the frame rate can be increased by using a pipelining readout that overlaps the sampling and readout periods of the row that receives light from the pixel. 

### 2.1. Ramp-Select Circuit Using Feedback Scheme

**Figure 4:**
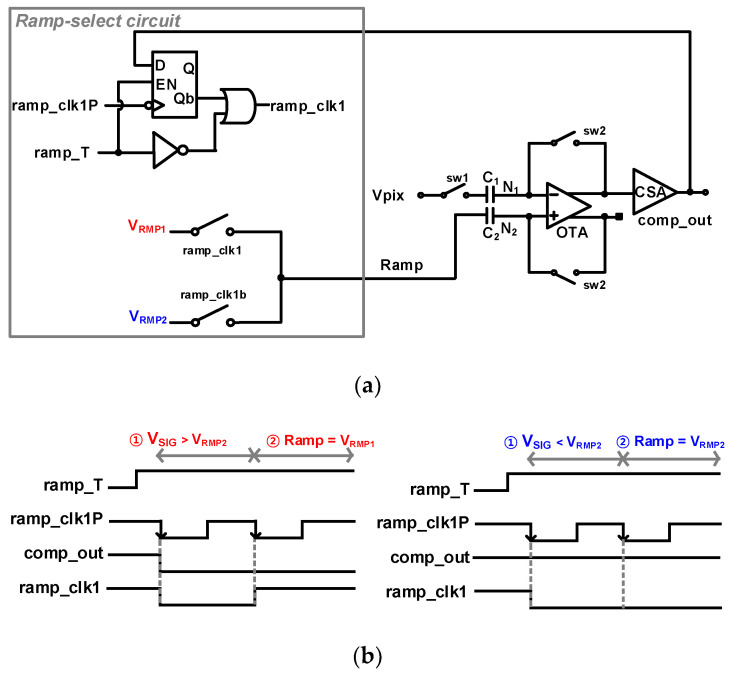
(**a**) Block diagram and (**b**) timing diagram of ramp-select circuit. Two timing diagrams are shown with different conditions of V_SIG_ to choose ramping signals between V_RMP1_ and V_RMP2_.

Unlike the existing CIS read-out method, it operates as a CMS under low light and CDS under high light; therefore, each of the two ramp signals must be appropriately selected. For this purpose, a ramp-select circuit was proposed and integrated into a conventional digital CDS (D-CDS) structure. Figure 4a shows a block diagram of a ramp–select circuit using D-CDS. For a four-transistor pixel, the reset voltage, V_RES_, is sampled first, followed by the signal voltage, V_SIG_. After auto-zeroing using C_1_ and C_2_, V_SIG_ and V_RMP2_ are sampled to compare which voltage is higher, and then the ramping signal is selected. With the proper ramping signal, the D-CDS for V_SIG_ sampling was performed. The period for selecting the ramping signals begins when the ramp_T signal in Figure 4b becomes ‘H’. When the negative-edge-triggered D flip-flop (N-ETDFF) is enabled by ramp_T, V_RMP2_ and V_SIG_ with 1/4 of ΔV_IN_ (=V_RES_ − V_SIG_) enter the input of the comparator, V_PIX_. Ramp_clk1P is the same as the system clock used for the ramp-select period between the reset and signal sampling periods. If the light entering the pixel has low illumination between 0 and 1/4 of ΔV_IN_, V_SIG_ is higher than V_RMP2_, and comp_out becomes ‘L’. The comparator output was fed back into the input of the N-ETDFF of the ramp-select circuit. When the second falling edge of ramp_clk1P occurs, ramp_clk1 becomes ‘H’ by the feedback comparator output, and V_RMP1_ is selected for the CMS operation. On the other hand, when the light entering the pixel is 1/4 to 1 of ΔV_IN_, V_RMP2_ is higher than V_SIG_, so comp_out is ‘H’. Therefore, the second falling edge of ramp_clk1P makes ramp_clk1 ‘L’, and V_RMP2_ is selected for the CDS operation.

### 2.2. Binary Calculation for HMS 

**Figure 5:**
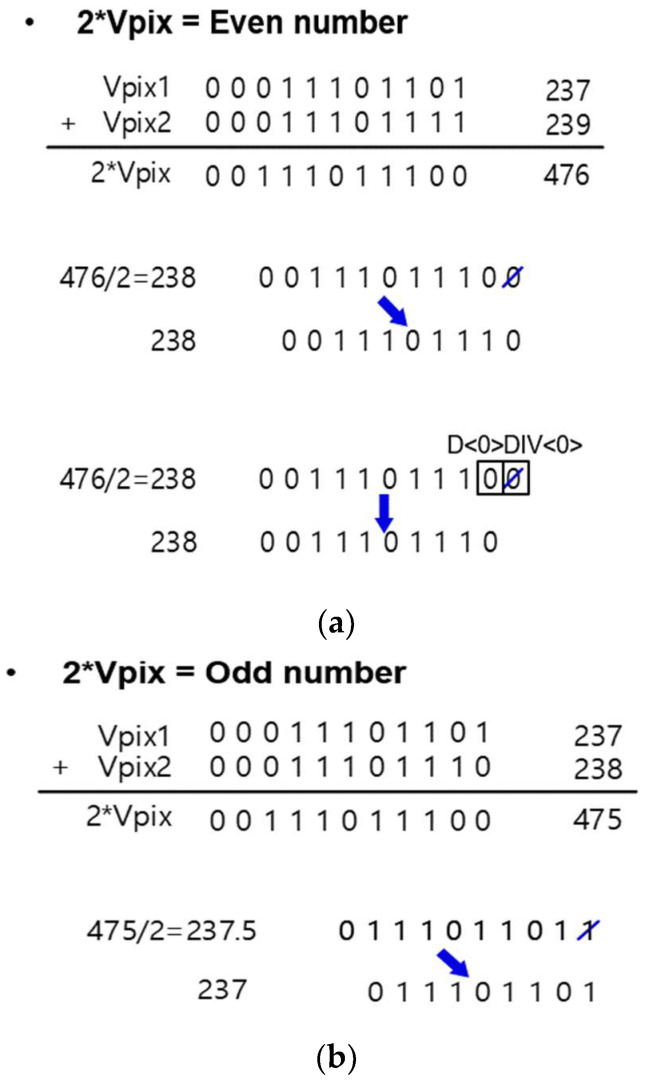
CMS operation for (**a**) doubled even output codes and (**b**) doubled odd output codes.

Unlike 1/4 to 1 of ΔV_IN_, which outputs a digital code according to the illuminance as is through single sampling, it requires sampling twice to reduce noise in low illuminance and calculates twice the digital code output. In the case of the existing CMS, an additional calculation circuit for *m* times digital code output is required when multi-sampling *m* times, but this can be calculated by adjusting the LSB owing to the nature of the digital code of binary numbers. During CMS operation under low illumination conditions, removing D<0>, which corresponds to the LSB of the digital code output twice, and shifting all bits from D<N> to D<N-1> is equivalent to dividing by two. Therefore, the digital code expressed in binary numbers can be outputted by dividing the double digital code by two by deleting the LSB and then shifting the code toward the LSB. Figure 5 shows two examples. For the even output codes, if the signal code for the first sampling is 237 and the signal code for the second sampling is 239, which is 237 + 239 = 476 code, the final output should be 476/2 = 238 code. As shown in Figure 5a, if we delete 0 corresponding to the LSB of the code corresponding to 476 and shift it, it is the same as the binary digital code corresponding to 238. Figure 5b illustrates the case of odd-output codes. The double digital code included 237 codes for the first sampling and 238 codes for the second sampling. In the case of the 237 + 238 = 475 code, each bit is shifted after discarding the LSB code; therefore, when 475/2 = 237.5, the decimal point is discarded to 237. This is similar to that of the corresponding binary digital code.

**Figure 6:**
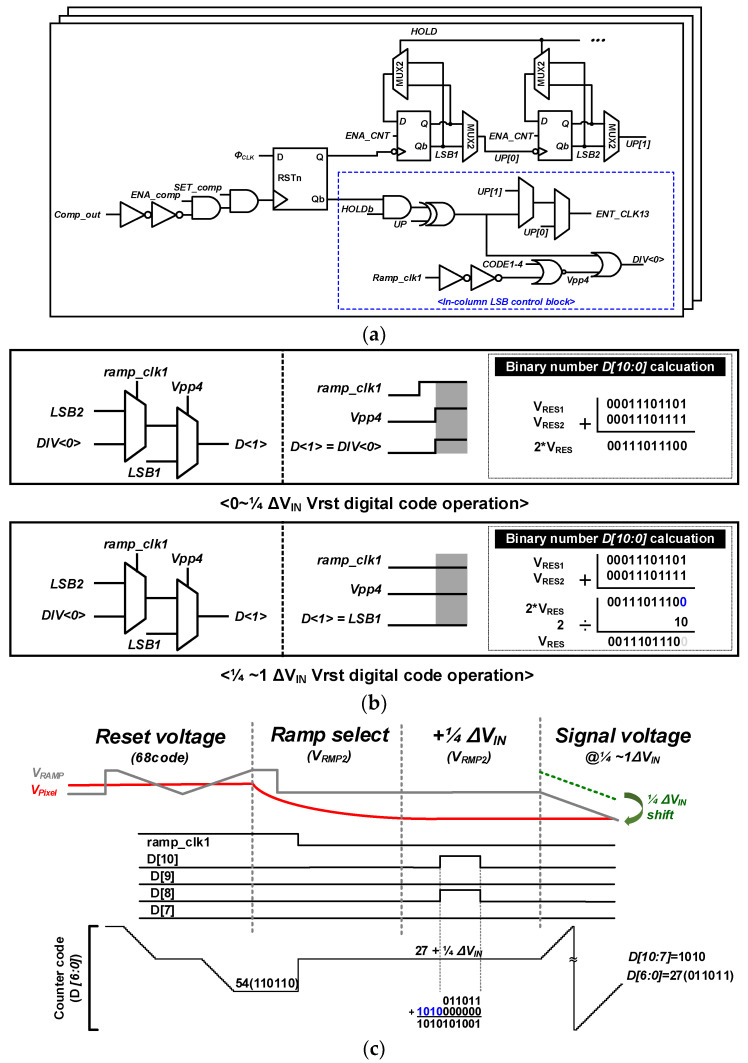
(**a**) Block diagram of in-column LSB control block, (**b**) operation of CMS, and (**c**) timing diagram of HMS with adaptive-dual gain ADC (1/4 < ΔV_IN_ < 1).

Figure 6a,b show the block diagram and operating principle of the column-parallel HMS circuit for controlling the LSB by illuminance in an adaptive-dual gain ADC. The HMS counter uses the conventional up-down counter (UDC) structure and the DIV<0> signal with the same period as the system clock (CLK) of 1 LSB. When the pixel output was less than 1/4 of ΔV_IN_, the reset voltage was doubled and the reset voltage was sampled twice, as shown in Figure 6b. The LSB clock period DIV<0> was determined through ramp_clk1 and Vpp4 signals using a multiplexer (MUX). When the pixel voltage is 1/4 to 1 of ΔV_IN_, the reset voltage doubled by V_RMP1_ must be divided into two before sampling the signal voltage. For this division operation, the LSB of the binary digital code was deleted and the digit was shifted by one place. For example, if the reset voltage is 54_(10)_, which is 110110_(2)_, then it becomes 27_(10)_ which is 11011_(2)_ by deleting and shifting the LSB by 0.

For signal sampling, V_RAMP_ starts from 1/4 of ΔV_IN_ of the pixel voltage. Therefore, unlike conventional UDC, a digital code corresponding to 1/4 of ΔV_IN_ is added. For example, as shown in Figure 6c, if the reset voltage is 8 bits and the signal voltage is 11 bits, then 10100000000_(2)_ should be added. Therefore, 10100000000_(2)_ from 1/4 of ΔV_IN_ and 11011_(2)_ during reset voltage sampling were added, resulting in D [10:0] = 10100011011_(2)_.

### 2.3. LSB Control Counter

**Figure 7:**
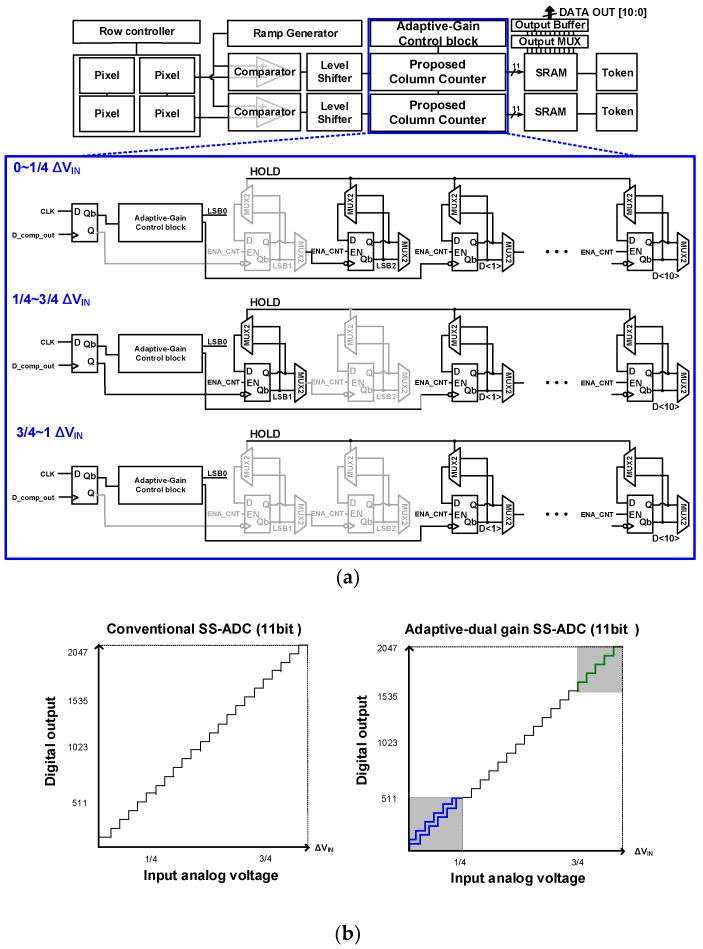
(**a**) Block diagram of the column counter and (**b**) the linearity comparison of conventional and the proposed adaptive-dual gain ADCs (blue line: CMS under low illumination, green line: ×2 using adaptive-dual gain ADC on the right figure).

Figure 7a shows a block diagram of the column counter, with unit counters operated for each ΔV_IN_. It consists of 12-unit counters for an 11-bit column counter, and an LSB is generated from LSB1, LSB2, and D<1>, which determines the digital gain at the three different frequencies. The control signal generated from the adaptive-dual gain control block determines the unit counter such that different digital gains are applied for each ΔV_IN_ range. This maintains the linearity of the ADC using the largest digital gain in a ramping signal with different analog gains at 1/4–3/4 of ΔV_IN_. Figure 7b compares the linearity of the conventional SS-ADC and adaptive-dual gain ADC. Similar to the conventional SS-ADC, the linearity of the proposed adaptive-dual gain ADC can be obtained in all ranges of ΔV_IN_.

## 3. Experiment Results

**Figure 8:**
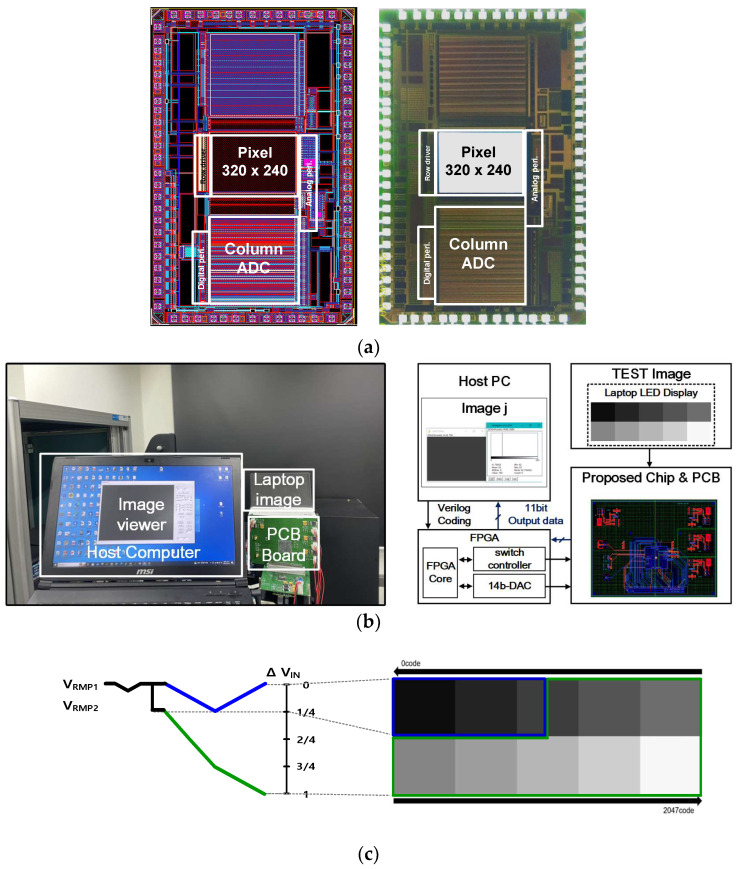
(**a**) A layout and chip microphotograph, (**b**) test environment, and (**c**) evaluated images with 2 ramps @ 0 to 1200 luxs.

The prototype chip of the proposed CIS was manufactured using a 0.11 μm process. Figure 8a shows a layout and a photograph of the prototype CIS chip occupying an area of 2.55 mm × 5.24 mm. The measurement environment for the proposed CIS is shown in Figure 8b, and the measurement sequence is as follows. An FPGA board, XEM3050 (Xilinx Spartan-3 FPGA Integration Module, Portland, OR, USA), was used to generate control signals to drive the image sensor on a computer. Using the ISE program, control signals were applied to the designated pins of the image sensor in Verilog. Verilog coding was completed, and the sensor output was sequentially read and confirmed using a computer’s image viewer. Xilinx’s Opal Kelly board was used to drive the FPGA based on the USB interface, and measurements were performed by checking the final image displayed in the image viewer. Figure 8c shows an image measured from the proposed CIS, indicating that all 11-bit codes are output through the ADC using two ramping signals for each illuminance. For the entire code, it was confirmed that the ramping signals were automatically selected according to illuminance.

### 3.1. Dark Random Noise

The relationship between the output code of the ADC that is finally output to calculate the conversion gain (CG) of the image sensor and CG is as follows:

(1) $$\left(\mathrm{Image}\;\mathrm{output}\;\mathrm{level}\right)=\left(\frac{1}{\mathrm{CG}}\right)\times \left(\mathrm{Incident}\;\mathrm{photon}\right)+\left(\mathrm{Image}\;\mathrm{offset}\;\mathrm{signal}\right)$$

The image offset signal is the signal level when no light is incident. In addition, the noise that appears in the image consists of temporal dark noise and shot noise. The equation is as follows:

(2) $$\left(\mathrm{Total}\;\mathrm{noise}\right)=\sqrt{{\left(\mathrm{temporal}\;\mathrm{dark}\;\mathrm{noise}\right)}^{2}+{\left(\mathrm{shot}\;\mathrm{noise}\right)}^{2}}\;$$

Temporal dark noise occurs when light is not incident; therefore, it can be expressed as a very small constant. The above equation can be summarized as follows:

(3) $${\left(\mathrm{Total}\;\mathrm{noise}\right)}^{2}=\left(\mathrm{number}\;\mathrm{of}\;\mathrm{photons}\right)=\left(\mathrm{CG}\times \mathrm{output}\;\mathrm{digital}\;\mathrm{value}\right)$$

**Figure 9:**
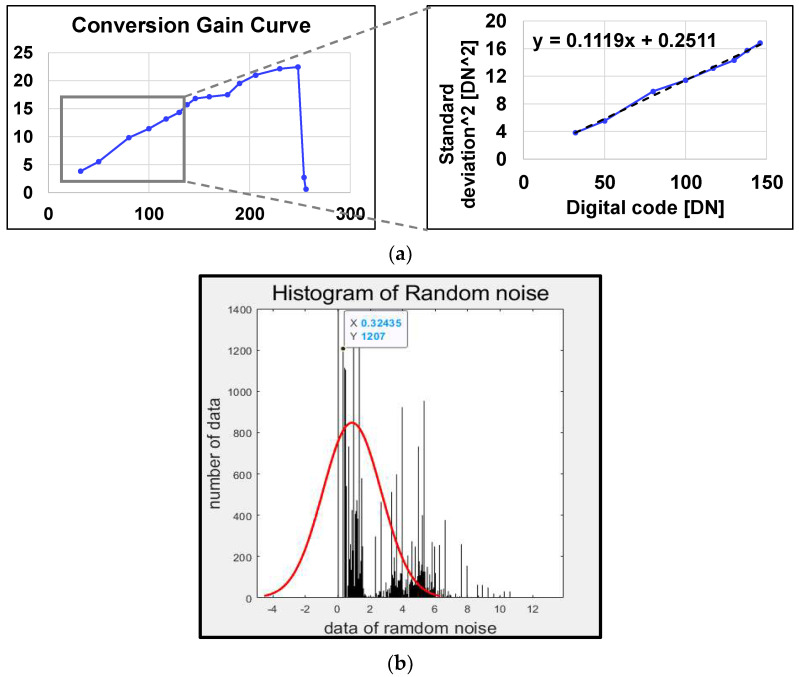
(**a**) Conversion gain graph and (**b**) DRN calculated with histogram using Matlab.

Figure 9 shows the measurement results for CG and DRN. In Figure 9a, CG is linear as DRN increases until the pixel is saturated by photons. Figure 9b shows the slope of the trend line CG = 0.1119 (DN/e^−^) and 43.71 (μV/e^−^), where DN is a digital number. DRN is the noise generated by the readout circuit, which is reflected in the output data and digital number (DN) during ADC measurement. The root-mean-square (RMS) DRN calculates the average DN caused by noise when no light enters a pixel. Because DN uses the standard deviation of the histogram, the image was measured several times and a value close to the average was obtained. Consequently, the RMS DRN of the proposed circuit is RN = 0.32435 (DN) and 2.9 (e^−^ rms).

### 3.2. Signal-to-Noise Ratio

Table 1 shows the signal-to-noise ratio (SNR) of the proposed SS-ADC with an HMS and the existing SS-ADC measured by dividing illuminance into three parts. According to Table 1, the proposed circuit shows a tendency for noise to increase as the light intensity increases. However, the SNR increases as the amount of light increases because the increase in the output digital code is larger. Compared to the existing SS-ADC, it increased by 53.4% at low illuminance and 15.31% at medium illuminance. Because the proposed circuit achieves a noise reduction effect through CMS under low illumination (@0 V~1/4 of ΔV_IN_), an SNR increase of 38.09% is obtained below 1/4 of ΔV_IN_.

Table 2 presents a performance comparison of low-noise CISs. Compared to the PMS in [11], the dark random noise in low light was reduced by up to 32% through dual sampling. The proposed CIS has up to 64% lower dark random noise than that in [13], which has low dark random noise with a CMS. In addition, we observed a lower figure of merit (FoM) of 38% at low illumination, corresponding to 0–1/4 of ΔV_IN_ compared to [13].

## 4. Conclusions

This paper explains the concept and structure of an SS-ADC with a noise reduction effect through CMS in low light, which achieves D-CDS with two ramping signals with different analog/digital gains. The measured image shows that two ramping signals with different ΔV_IN_ values can be applied by automatically detecting the amount of incident light using a ramp-select circuit. The measurement results of the prototype sensor show that image visibility, dark random noise reduction, and SNR are improved through the HMS, especially in low-illuminance areas. As a result, the proposed CIS is expected to be used in various applications such as automotive CIS and object detection.

## Figures and Tables

**Table 1:** Measured output (mV), noise (mV), SNR (dB) with different illumination (Lux).

Illumination	Output	Noise	SNR	SS-ADC SNR
340	95.40	2.18	32.80	21.38
900	275.39	6.11	33.06	28.68
1280	466.64	8.87	34.42	28.71

**Table 2:** Comparison of performance characteristics of the proposed CIS with other works.

	[11]	[13]	This Work
Technology (nm)	90	90	110
Image Resolution	8 M	960 × 720	320 × 240
Pixel Size (μm^2^)	1.4 × 1.4	1.4 × 1.4	3.25 × 3.25
ADC Column Pitch (μm)	2.8 (even/odd readout)	2.8 (even/odd readout)	3.25 (even readout)
ADC Resolution (bit)	10	10	11
System CLK (MHz)	130	50	12.5
Random Noise (V_RMS_)	336 μ	472 μ	80.39 μ 169.69 μ
Total Power (mV)	280	28	5.349
FoM (V_RMS_·W/FPS·# of Pixels)	N/A	542.8 μ @dark	336 μ @dark 710.15 μ @bright

## Data Availability

The datasets generated from the current study are available from the corresponding author upon reasonable request.
